# Supplementary material for: The bone bridge significantly affects the decrease in bone mineral density measured with quantitative computed tomography in ankylosing spondylitis
Source: PLoS One. 2021 Apr 16;16(4):e0249578. doi: 10.1371/journal.pone.0249578 (PMC8051772; doi:10.1371/journal.pone.0249578)
Supplement: Attachment — Submitted filename: Response to Reviewers.docx [file pone.0249578.s001.docx]

**To the Editor-in-Chief, PLOS ONE**

**Dear Editor:**

First of all, thank you very much for your consideration of publication of this paper in PLOS ONE. We made efforts to follow your recommendations and suggestions as best as we could. We addressed several important concerns that the reviewers brought up and tried to revise the manuscript appropriately. We hope that our response and revision can alleviate the reviewers’ concerns.

Thank you once again for your time and consideration.

Sincerely Yours,

Sang-Hoon Lee, MD

The review panel was enthusiastic about this manuscript and had overall minor concerns about addressing non-clinical factors that may contribute to osteoporosis in AS patients. Although the panel is in agreement that qCT is a better method, the authors also need to reinforce the reason to not pursue DXA, since it is still considered the clinical gold standard for bone mineral measurements, and the audience of the Journal may look for that link between methods. Lastly, a power analysis also is deemed necessary to lend support to the statistical findings.

> Thank you for your thoughtful comment. We have mentioned whether DXA use is not appropriate in AS patients. In AS, it is known that BMD is overestimated due to ossification of ligament around the vertebral body (reference #23). In the previous study, when using DXA to diagnose osteoporosis, fewer patients were diagnosed with osteoporosis than when measured by QCT. Diagnosed (reference #28). Although DXA is still the most used diagnostic method for osteoporosis, it should be considered as the diagnosis through DXA may not be as accurate as in AS. I have marked the sentences related to the first page of the discussion.

We also performed a power analysis in response to your comment. As a result of post hoc analysis on the 47 patients used in this study, the power was 0.96 at ρ0 = -0.501, α = 0.05. This is added at the end of the 'Correlation between bone bridge and QCT BMD' part of the result.

**Reviewers' comments to the author:**
**Reviewer #1:**

This is a well written article examining the association between bone bridging, lumbar ROM and QCT assessed BMD. Using retrospective data the authors are able to effectively demonstrate a negative correlation between QCT BMD, bone bridging, and lumbar ROM. The discussion provides a good frame work for the utilization of this association. Osteoporosis in AS patients is likely multifactorial based on the chronic inflammatory state as well as decreasing physical activity (lumbar ROM).

The limitation of this article is the relatively small data set. For example, although statistically insignificant in their evaluation, the use of TNF inhibitors differed by about 20% between the normal and low BMD groups, begging the question, would these medications have some significance in a larger data set.

> Thank you for your thoughtful comment. We agree with your point. As there have been previous studies showing that anti-TNF therapy slows down generalized bone loss in AS patients, an accurate analysis is essential that there is no difference in anti-TNF therapy between the two groups when the sample size increases. The content of the positive effect of the TNF inhibitor was mentioned in the text.

This article is also limited in terms of the use of QCT for the evaluation of BMD in this patient population. Although QCT appears to correlate with bone bridging and lumbar ROM, without a DXA value to compare to as an internal control (from both the hip and lumbar spine), I don’t think the rationale for utilization of QCT over DXA is entirely supported.

> Thank you for your thoughtful comment. As mentioned in the text, if there was a direct comparable DXA value, it would have been a sufficient basis for performing QCT rather than DXA in AS patients. However, it is difficult to use both methods when measuring BMD in one patient due to problems such as insurance and cost of DXA test. In the study we cited as reference # 28, it was confirmed that BMD is overestimated in DXA when compared to QCT in patients with AS due to spinal involvement. In this study, it was mentioned in a limitation that the analysis of BMD was not performed in both DXA and QCT methods.

Overall, this is an interesting article that introduces the interplay between bone bridging, physical activity (ROM), and osteoporosis in the AS population. Although it does not provide any detail of causality in regards to this interplay, this introduces a new avenue for future research on this topic.

**Reviewer #2:**

The purpose of this study was to determine whether the formation of a bone bridge correlates with low BMD measured with QCT in patients with AS.

Authors concluded that the bone bridge significantly affects the decrease in bone density in patients with AS.

This study could support the usefulness of early identification of bone loss to prevent spinal radiographic progression of AS.

However, pathogenesis of osteoporosis could be multifactorial and bony bridge might not be the main pathology of osteoporosis in AS patients. There could be possibilities that osteoporosis is more related to socioeconomic status, occupational status, and disability caused by spinal malalignment. Author should describe this.

> Thank you for your thoughtful comment. We agree with your point. In the baseline characteristics analysis, one of our limitations is that the factors known to cause osteoporosis were not sufficiently identified. In response to this comment, we emphasized by adding content to the text.
